# Proteinuria With Functionally Normal Kidneys: A Hidden Clue to the Diagnosis of Diffuse Large B-cell Lymphoma (DLBCL) With Secondary Glomerular Infiltration

**DOI:** 10.7759/cureus.98124

**Published:** 2025-11-30

**Authors:** Bineetha Babu Satheendranath, Deena John, Vimal Kumar, Ahmed Raza, Raman Verma

**Affiliations:** 1 Respiratory Medicine, University Hospitals of Leicester NHS Trust, Leicester, GBR

**Keywords:** dlbcl with renal involvement, glomerular infiltration in dlbcl, lymphoma responded to pola chp regimen, nephrotic syndrome in lymphoma, proteinuria without renal dysfunction in dlbcl

## Abstract

Diffuse large B-cell lymphoma (DLBCL) is an aggressive, rapidly progressive type of non-Hodgkin lymphoma that arises from mature B lymphocytes and is characterized by diffuse involvement of lymph nodes as well as possible extranodal tissues. It typically presents with fever, night sweats, weight loss and lymph node enlargement. While extranodal organ involvement is relatively common, the renal involvement remains a rare and under-recognized manifestation. We report the case of a 66-year-old female patient initially admitted with lower respiratory tract infection (LRTI), who was later diagnosed with DLBCL involving the kidneys. The fever began a week before admission, and despite receiving appropriate treatment for LRTI, she continued to spike intermittent fevers. Clinical assessment revealed pedal oedema, hypoalbuminemia and nephrotic range proteinuria, with preserved renal function. A fluorodeoxyglucose positron emission tomography (FDG PET) scan demonstrated metabolically active cervical lymphadenopathy, and subsequent biopsies of kidney and cervical lymph node confirmed the diagnosis of DLBCL. Timely initiation of the polatuzumab vedotin, rituximab, cyclophosphamide, doxorubicin, and prednisolone (Pola-R-CHP) chemotherapy regimen led to significant clinical improvement within six weeks of diagnosis. This case highlights the importance of recognizing renal involvement in atypical presentations of DLBCL, emphasizing the need for clinical vigilance to facilitate early diagnosis, timely management, and improved patient outcomes.

## Introduction

Diffuse large B cell lymphoma (DLBCL) originates from mature B lymphocytes and represents the most common pathological subtype of non-Hodgkin lymphoma (NHL) [1]. It accounts for around 40% of all non-Hodgkin lymphomas worldwide [2]. Although renal involvement is found in approximately 45% of lymphoma cases at autopsy (ranging from 30% to 60%), it is diagnosed by CT imaging only in around 5% of patients. The kidneys are the most frequently affected abdominal organ in lymphoma. Most cases involve B-cell non-Hodgkin lymphoma, while primary renal lymphoma is uncommon (<1%). Renal involvement in Hodgkin lymphoma is also rare (<1%) [3].

There are several explanations for renal involvement in lymphomas, with the most common being direct infiltration by lymphoma cells. Renal manifestations related to lymphoma often go undetected due to lack of specific clinical signs. Biopsy typically demonstrates tubular occlusions caused by diffuse infiltration of malignant lymphocytes, while the tubules and glomeruli remain histologically normal [4]. This infiltration occurs mainly in one of two patterns: tubulointerstitial involvement, which typically manifests as bilateral kidney enlargement or a renal mass [4], and glomerular invasion, in which malignant cells obstruct the capillary lumen, potentially causing acute kidney injury in some instances [5]. The nephrotic syndrome in lymphoma may either occur because of minimal change disease induced by cytokine-mediated increases in glomerular membrane permeability or due to invasion of glomeruli by lymphomatous cells, leading to effacement of foot processes [5]. Minimal change nephrotic syndrome (MCNS) is a common glomerular complication of Hodgkin lymphoma and occurs less frequently in non-Hodgkin lymphoma. Both idiopathic and lymphoma-associated MCNS are believed to arise from an unidentified soluble permeability factor that increases glomerular capillary permeability, causing albuminuria. In lymphoma-associated MCNS, this factor is considered paraneoplastic. Both forms exhibit immune features: T cells favor T-helper type 2 (Th2) differentiation, with upregulated c-Maf and c-Maf-inducing protein (c-mip) and reduced IL-12 receptor expression. The combined effects of Th2 dominance and regulatory T-cell dysfunction lead to cytokine overproduction, increasing glomerular basement membrane permeability. Focal segmental glomerulosclerosis (FSGS) has been observed in both Hodgkin and non-Hodgkin lymphoma patients and is considered a potential progression from the podocyte injury seen in minimal change disease (MCD), representing a later stage of lymphoma-associated glomerular damage. Amyloidosis, though rare, occurs more commonly in Hodgkin lymphoma than in non-Hodgkin lymphoma and presents with massive proteinuria, hypoalbuminemia, anasarca, and renal dysfunction and hypotension [4]. Immune-mediated renal injury in lymphoma may result from deposition of the monoclonal light and/or heavy chains (paraproteins), the production of autoantibodies, the formation of immune complexes and cryoglobulinemia [1,6,7]. Membranous and membranoproliferative glomerulonephritis, and occasionally crescentic glomerulonephritis, have been linked to both Hodgkin and non-Hodgkin lymphomas, with lymphoma-related membranous glomerulonephritis showing antibody deposition along the glomerular basement membrane [4]. This case describes nephrotic syndrome with normal renal function as an initial and atypical presentation, which ultimately led to the diagnosis of DLBCL with glomerular infiltration.

## Case presentation

A 66-year-old woman with a history of polymyalgia rheumatica, for which she had been receiving long-term prednisolone 15 mg once daily for the preceding three months, presented in May 2025 with a one-week history of fever, fatigue, and shortness of breath. On admission, the patient was tachypneic at rest and hypoxic, with an oxygen saturation (SpO₂) of 96% on 1 liter of oxygen. Blood pressure was 155/87 mmHg, temperature 38°C, heart rate 95 beats per minute, and respiratory rate 20 breaths per minute. Initial blood investigations showed white cell count (WCC ) of 5.8 (10⁹/L), neutrophils of 4.72 (10⁹/L), eosinophil count of 0.01 (10⁹/L), haemoglobin of 92g/L, C- reactive protein of 138 mg/L, sodium of 132 mmol/L, potassium of 4.2 mmol/L, creatinine of 48 µmol/L, estimated glomerular filtration rate (eGFR) >90 ml/min/1.73 m² and serum albumin was 27 g/L (Table 1). Respiratory virus PCR panel detected influenza A virus RNA. She was diagnosed with lower respiratory tract infection due to influenza A with suspected superimposed bacterial infection and was commenced on a five-day course of co-amoxiclav, doxycycline, and oseltamivir. Despite appropriate antimicrobial and antiviral therapy, she continued to experience on-and-off fevers. In the subsequent days, urine microscopy showed no evidence of infection, with no casts or dysmorphic red cells. Urine, sputum and blood cultures did not show any growth and no alternative foci of infection were identified clinically. Persistent, non-resolving, new-onset bilateral pitting pedal oedema, extending to the middle of the shin, was also noted at the same time. Meanwhile, the autoimmune screen, including antinuclear antibody (ANA), antineutrophilic cytoplasmic antibody (ANCA), serum paraprotein analysis, and complement levels, and blood-borne virus screen yielded negative results. Repeat blood tests revealed hypoalbuminaemia (26 g/L) with normal renal function and further urine analysis demonstrated nephrotic-range proteinuria. The urine protein profile was as follows: urine random protein of 1.67 g/L, urine albumin of 757.90 mg/L, urine random creatinine of 2.9 mmol/L, urine protein creatinine ratio of 575.9 mg/mmol, urine albumin creatinine ratio of 261mg/mmol. A fluorodeoxyglucose positron emission tomography (FDG PET) scan was also requested in view of persistent fever for more than three weeks to evaluate for underlying malignancy.

**Table 1:** Laboratory Investigations Blood investigations on admission, showing normal kidney function and hypoalbuminemia. eGFR: estimated glomerular filtration rate.

Tests	Results	Reference range
White Cell Count	5.8 (10⁹/L)	4.0-11.0 (10⁹/L)
Haemoglobin	92 g/L	115-165 g/L
Neutrophil count	4.72 (10⁹/L)	1.50-7.50 (10⁹/L)
Eosinophil count	0.01 (10⁹/L)	0.04-0.40 (10⁹/L)
C-reactive protein	138 mg/L	0-10 mg/L
Sodium	132 mmol/L	133-146 mmol/L
Potassium	4.2 mmol/L	3.5-5.3 mmol/L
Creatinine	48 µmol/L	60-120 µmol/L
eGFR	>90 ml/min/1.73 m²	
Albumin	27 g/L	35-50 g/L

In view of nephrotic-range proteinuria consistent with nephrotic syndrome, an urgent renal biopsy was scheduled around the fourth week of admission. The renal biopsy specimen consisted of a core of renal cortex with approximately 25 glomeruli in a single plane of section. All of the glomeruli showed global or segmental involvement by atypical pleomorphic cells within the capillary loops, resulting in luminal occlusion. Similar cells were also identified focally within the interstitium. There was no mesangial matrix expansion, basement membrane duplication, subepithelial spike formation, segmental sclerosis, crescent formation or fibrinoid necrosis. There was no evidence of acute tubular injury, tubulointerstitial nephritis, or tubular casts. Congo-red staining was negative for amyloid. Immunofluorescence on four glomeruli was negative for IgG, IgA, IgM, C1q, C3, and IgG4 with no features of light chain restriction. Electron microscopy revealed no electron-dense materials but demonstrated widespread foot process effacement, likely secondary to intraglomerular obstruction. Overall, the biopsy revealed no intrinsic renal pathology on light microscopy, immunofluorescence or electron microscopy. On immunohistochemistry, the atypical cells were positive for CD45, CD20, and PAX5, confirming a B-cell phenotype. There was co-expression of BCL2, BCL6, MUM1, and c-MYC (>40% of cells positive). The Ki-67 proliferation index was high (>60%). The immunophenotypic profile favored an activated B-cell phenotype with apparent double expression of BCL2 and c-MYC. Fluorescent in situ hybridization (FISH) testing was not performed.

**Figure 1:**
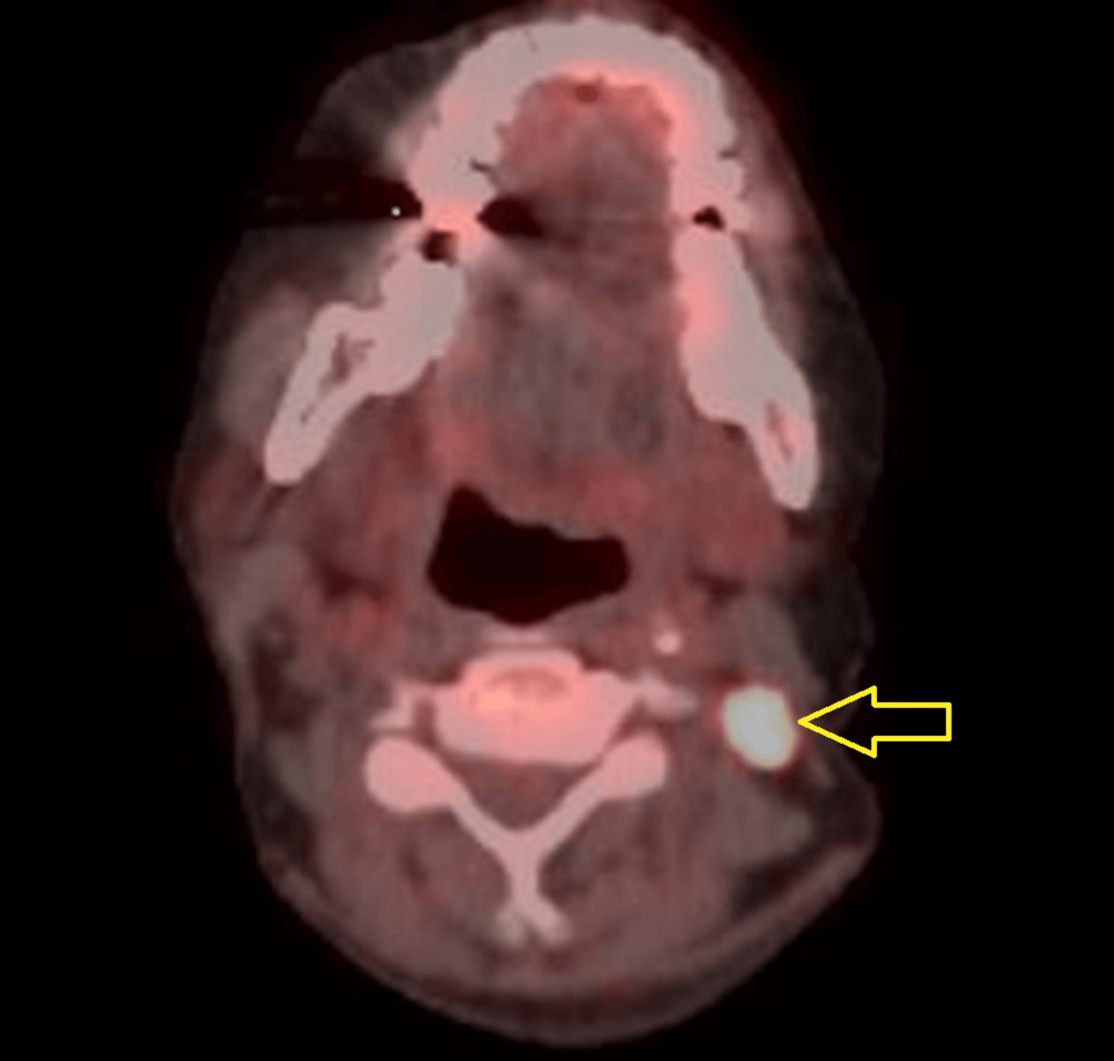
Fluorodeoxyglucose positron emission tomography (FDG PET) scan before treatment Fused axial PET scan demonstrating increased FDG uptake in the left cervical lymph node prior to treatment initiation (arrow).

As a concurrent study, FDG PET/CT demonstrated metabolically active lymphadenopathy. Multiple intensely FDG-avid left cervical lymph nodes were noted at levels II, III, and IV, the largest in level IIB measuring 10 mm with an SUV max of 20.9 (Figure 1). No right cervical, abdominal, or pelvic lymphadenopathy was identified. The spleen was normal in size and metabolic activity, and no suspicious pulmonary lesions were detected. The skeletal muscles, aerodigestive tract and solid abdominal organs appeared unremarkable, and there were no FDG-avid bone lesions. The cervical lymph node biopsy was also performed, which revealed diffuse infiltration by large atypical lymphoid blast cells exhibiting nuclear pleomorphism, irregular nuclear contours, and prominent nucleoli. Abundant mitotic activity and apoptotic debris were present. A panel of immunohistochemistry was undertaken on the lymph node biopsy sample, which confirmed atypical blast cells with positive expression for CD20, CD79 and PAX5, confirming a B-cell lineage. There was also co-expression for BCL2, MUM1 (>30%), and c-MYC (~40%). The Ki-67 proliferation index was high, approximately 70%. Although double-expressor lymphoma (DEL) is considered a group of DLBCL characterized by overexpression of c-MYC (≥40%) and BCL2 (≥50%) by immunohistochemistry, the biopsy in this patient showed co-expression of BCL2 and c-MYC, indicating the aggressive nature of the disease. These biopsies confirmed activated B-cell type diffuse large B-cell lymphoma with secondary renal infiltration resulting in nephrotic syndrome.

**Figure 2:**
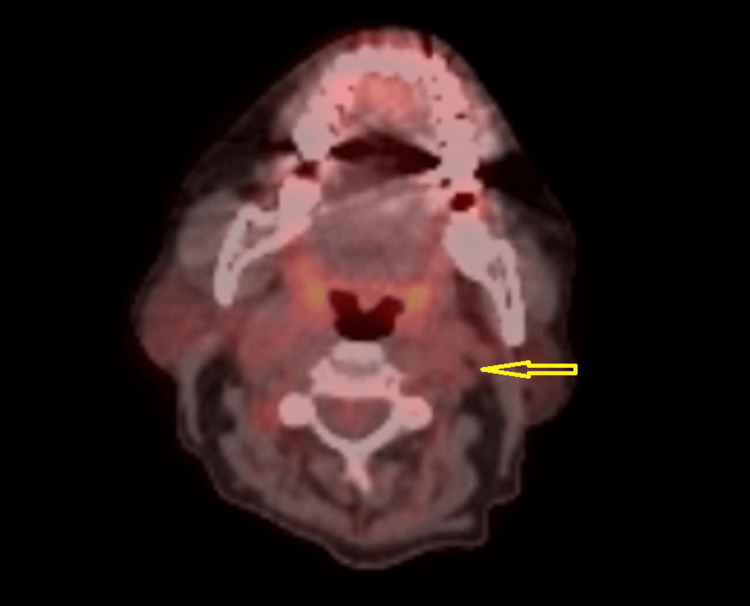
Fluorodeoxyglucose positron emission tomography (FDG PET) scan after treatment Fused axial PET view, performed eight weeks after the first cycle, showing complete resolution of left cervical lymphadenopathy following two cycles of chemotherapy (arrow).

The case was discussed at the lymphoma multidisciplinary team (MDT) meeting, where a plan was made to commence polatuzumab vedotin, rituximab, cyclophosphamide, doxorubicin, and prednisolone (Pola-R-CHP) chemotherapy regimen. She was subsequently transferred to the haematology team and initiated on chemotherapy. Her temperature spikes and oxygen requirements resolved within one week of the first cycle. As she tolerated the initial cycle well, the decision was made to continue with the subsequent cycles. She was thereafter discharged, with plans for the remaining treatment to be delivered in the outpatient setting. The second cycle of Pola-R-CHP was administered approximately three weeks after the first. A repeat FDG PET scan performed around three weeks after the second cycle demonstrated complete resolution of the right cervical lymphadenopathy, with a Deauville score of 1 (Figure 2). The repeat urine protein profile also showed resolution of proteinuria (Table 2). The random urine protein level was <0.06 g/L, and the random urine creatinine was 2.3 mmol/L; therefore, the protein-creatinine ratio could not be calculated. The remaining chemotherapy cycles were administered at two-to-three-week intervals, and at the time of writing, she has completed a total of six cycles. Interim reviews and the assessment after the sixth cycle confirmed sustained clinical improvement, with no significant pedal oedema or fever. A further PET-CT scan is planned in the coming weeks, with follow-up arranged to review the results.

**Table 2:** Comparison of urine protein parameters pre- and post-treatment Urinary protein levels before treatment and after two cycles of chemotherapy (eight weeks from initiation), showing normalization of proteinuria.

Urine protein profile	Before treatment	After treatment	Reference range
Protein/Creatinine Ratio	575.9 mg/mmol	Due to low protein, unable to calculate ratio	0 – 30 mg/mmol
Random Protein	1.67 g/L	<0.06 g/L	0.01 – 0.14 g/L
Urine Albumin/Creatinine Ratio	261 mg/mmol creatinine	Nil	0.0 – 3.5 mg/mmol creatinine
Urine Albumin	757.90 mg/L	Nil	0 – 15 mg/L
Random Creatinine	2.9 mmol/L	2.3 mmol/L	-

## Discussion

Although renal involvement occurs frequently in lymphomas, it typically remains subclinical unless renal biopsy is performed or renal failure develops. Even when present, it is often underrecognized and may remain undiagnosed until postmortem examination [4]. While ultrasound is the first-line radiological investigation, CT scan is the preferred imaging modality when lymphoma is suspected. MRI scan can also be used. FDG PET CT scan is considered the gold standard for lymphoma staging and detecting disease recurrence, as its ability to assess tumor metabolic activity enhances both its sensitivity and specificity [8].

In lymphoid malignancies, particularly in Hodgkin lymphoma, nephrotic syndrome is commonly associated with a cytokine-induced minimal change disease, while in non-Hodgkin lymphoma, involving DLBCL, nephrotic syndrome is often linked to glomerular pathologies resulting from immune complex deposition [6]. Virginie Royal et al. (2025) state that paraneoplastic glomerular diseases are driven by tumor-secreted factors, primarily tumor antigens rather than direct renal invasion, and although their pathogenesis is not fully understood, they are believed to result from an immune response to these antigens, making recognition crucial as their management differs and can significantly influence treatment of the underlying malignancy [7]. In our patient, urine microscopy showed no evidence of glomerulonephritis, the autoimmune screen was negative for autoantibodies, and serum paraprotein levels were normal. Renal biopsy revealed no immune complex deposition or intrinsic renal pathology, and amyloid staining was negative, effectively excluding direct immune-mediated renal injury. Despite these findings, the biopsy confirmed the presence of lymphomatous cells within the glomeruli causing foot process effacement, making cytokine-mediated minimal change disease unlikely. A case has been previously reported by Pothen et al. (2019), where nephrotic syndrome with normal kidney function was described in association with intravascular large B-cell lymphoma, a rare extranodal subtype of DLBCL [5]. Additionally, cases have reported renal infiltration by DLBCL leading to acute kidney injury [9,10], DLBCL presenting with Fanconi syndrome [11], and glomerular invasion by lymphoma associated with proteinuria and renal impairment [6]. In contrast, our case describes a patient with normal renal function presenting with features mimicking nephrotic syndrome, which was ultimately revealed to be systemic DLBCL with secondary nephrotic syndrome, distinguished by the absence of immune complex deposition and the presence of malignant cells within the glomeruli causing mechanical obstruction and massive proteinuria. Misdiagnosis as primary nephrotic syndrome would have been possible given the patient’s normal kidney function, nephrotic-range proteinuria, and pedal oedema, which are classic features of primary glomerular disease.

In cases with unexplained proteinuria and systemic symptoms, timely biopsy is essential in differentiating the underlying renal pathology. In our case, the renal biopsy played a pivotal role in establishing the definitive diagnosis, particularly given the patient’s preserved renal function. The absence of amyloid deposition, clear immunophenotypic markers consistent with an activated B-cell lineage and supportive lymph node biopsy findings strengthened the diagnostic evidence of systemic DLBCL. Despite the aggressive nature of the disease, indicated by Ki-67 proliferation index of around 70% and double expression of BCL2 and c-MYC, the Pola-R-CHP regimen - a newer therapeutic combination - achieved rapid clinical and biochemical remission, including resolution of proteinuria and complete metabolic response on PET scan, reinforcing the therapeutic potential of this regimen in high-risk DLBCL with extranodal involvement.

## Conclusions

Although nephrotic syndrome is an atypical presentation associated with lymphoma, this case reinforces the need for a high index of suspicion and comprehensive evaluation in cases of unexplained proteinuria. As glomerular and interstitial patterns of involvement differ between Hodgkin and non-Hodgkin lymphomas, and the mechanisms underlying nephrotic syndrome vary by lymphoma subtype, renal manifestations can be diagnostically challenging. Meticulous clinical assessment, timely renal biopsy, and multidisciplinary collaboration are therefore essential for accurate diagnosis and guiding appropriate therapy. This case reinforces the importance of considering malignancy in patients with nephrotic-range proteinuria without intrinsic renal pathology and contributes to the growing literature on the diverse and often subtle presentations of DLBCL.
